# Detection of Retinitis Pigmentosa by Differential Interference Contrast Microscopy

**DOI:** 10.1371/journal.pone.0097170

**Published:** 2014-05-08

**Authors:** Juyeong Oh, Seok Hwan Kim, Yu Jeong Kim, Hyunho Lee, Joon Hyong Cho, Young Ho Cho, Chul-Ki Kim, Taik Jin Lee, Seok Lee, Ki Ho Park, Hyeong Gon Yu, Hyuk-jae Lee, Seong Chan Jun, Jae Hun Kim

**Affiliations:** 1 Sensor System Research Center, Korea Institute of Science and Technology (KIST), Seoul, Republic of Korea; 2 School of Mechanical Engineering, Yonsei University, Seoul, Republic of Korea; 3 Department of Ophthalmology, Seoul National University Boramae Hospital, Seoul, Republic of Korea; 4 Department of Ophthalmology, Seoul National University Hospital, Seoul, Republic of Korea; 5 Department of Nano & Electronic Physics, Kookmin University, Seoul, Republic of Korea; University of Florida, United States of America

## Abstract

Differential interference contrast microscopy is designed to image unstained and transparent specimens by enhancing the contrast resulting from the Nomarski prism-effected optical path difference. Retinitis pigmentosa, one of the most common inherited retinal diseases, is characterized by progressive loss of photoreceptors. In this study, Differential interference contrast microscopy was evaluated as a new and simple application for observation of the retinal photoreceptor layer and retinitis pigmentosa diagnostics and monitoring. Retinal tissues of Royal College of Surgeons rats and retinal-degeneration mice, both well-established animal models for the disease, were prepared as flatmounts and histological sections representing different elapsed times since the occurrence of the disease. Under the microscopy, the retinal flatmounts showed that the mosaic pattern of the photoreceptor layer was irregular and partly collapsed at the early stage of retinitis pigmentosa, and, by the advanced stage, amorphous. The histological sections, similarly, showed thinning of the photoreceptor layer at the early stage and loss of the outer nuclear layer by the advanced stage. To count and compare the number of photoreceptors in the normal and early-retinitis pigmentosa-stage tissues, an automated cell-counting program designed with MATLAB, a numerical computing language, using a morphological reconstruction method, was applied to the differential interference contrast microscopic images. The number of cells significantly decreased, on average, from 282 to 143 cells for the Royal College of Surgeons rats and from 255 to 170 for the retinal-degeneration mouse. We successfully demonstrated the potential of the differential interference contrast microscopy technique’s application to the diagnosis and monitoring of RP.

## Introduction

The light-sensing retinal tissue is composed of specially functionalized cell layers including the ganglion cell layer (GCL), the bipolar/horizontal cell layers, and the photoreceptor cell layer. The outer retinal layer, consisting of the outer plexiform layer (OPL), the outer nuclear layer (ONL), and the photoreceptor layer, detects, by means of numerous rods and cones, contrast and color, respectively. The photoreceptors for the initial sensing of light receive the light information of visual objects and transfer signals to retinal ganglion cells and optic nerves in the brain. Photoreceptor degeneration causes retinitis pigmentosa (RP), one of the most common inherited ophthalmologic diseases [1]. The histological changes effected by RP initially are detected as shortening of the photoreceptor outer segments and loss of photoreceptors [2]. In contrast to the uniformly dispersed mosaic pattern of normal photoreceptor cells, RP photoreceptor cells are displaced and non-uniformly distributed [3]. As RP progresses, whole outer layers collapse into a debris layer between the inner nuclear layer (INL) and the retinal pigment epithelium (RPE) [4].

Patients in the early stage of RP usually suffer from night blindness since primary RP incurs rod degeneration. Night blindness symptoms worsen as the disease progresses, followed by constriction of vision and, eventually, central vision loss [5]. Because RP symptoms occur after substantial photoreceptor loss [6], visualization and imaging of individual photoreceptors, particularly in real time and *in vivo*, can be useful for early diagnosis. Although there is no approved treatment for RP, vitamin A and docosahexaenoic acid have been reported to delay its progress [7], [8]. Early diagnosis of RP, moreover, can facilitate genetic counseling and future-disability planning. Therefore, many attempts to detect early RP have been made with various apparatuses such as optical coherence tomography (OCT) [9], magnetic resonance imaging (MRI) [4], and adaptive optics scanning laser ophthalmoscopy [10]. However, OCT and MRI measure photoreceptor layer thickness rather than perform cell-level imaging, rendering them ineffectual for detection of early RP-induced change. Adaptive optics scanning laser ophthalmoscopy can visualize individual photoreceptors in real time *in vivo*, though imaging of rod cells remains a challenge [10].

Differential interference contrast (DIC) microscopy is designed to image unstained, transparent specimens, enhancing the contrast resulting from the optical path difference between two beams that are split from the Nomarski prism. In the present study, we set up a DIC microscopy system and observed photoreceptor cells with RP at the cell level.

## Materials and Methods

### Differential Interference Contrast Microscopy System

**Figure 1:**
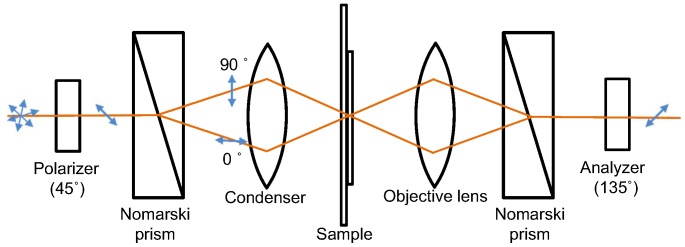
Experimental DIC setup. From the left, a polarizer at an angle of 45°, a Nomarski prism, a condenser, a sample, an objective lens, a Nomarski prism, and an analyzer at 135° are aligned. After passing through the first prism, the illuminated beam is split into two different polarizations. Retardation due to the thickness variation of samples generates interference after the analyzer.

The DIC microscopy system used in our laboratory is comprised of a polarizer, Nomarski prisms, a condenser, an objective lens, and an analyzer (Fig. 1; for additional details, see [11]). Light polarized to 90 degrees by the polarizer is split by a birefringent material. Each beam has orthogonal polarizations of 45, 135 degrees. After passing through a sample, the two beams move in the same path; then, they interfere when the analyzer, at 0 degrees, matches their polarization. The two beams pass through different refractive indices and thickness levels, and generate interference after the analyzer. Since interference occurs between ordinary and extraordinary wavefronts in the image plane without retardation, extinction at a broad intensity range can be obtained. The Nomarski prisms used in the present experimentation have a converging point 41.6 mm from the outer surface of the prism. Because of the advantage of the DIC for observing transparent samples in three dimensions (due to the interference contrast), a variety of specimens have been applied such as asbestos detection [11], surface roughness measurement [12], endocytosis observation of nanoparticles penetrating into human lung cells [13], and monitoring of blood flow passing through microfluidic channels [14].

### Animals and Retina Preparation

The use of animals in this study was approved by the Animal Research Committee of Seoul National University Hospital, and complied with the regulations of the Association for Research in Vision and Ophthalmology’s Statement for the Use of Animals in Ophthalmic and Vision Research. Royal College of Surgeons (RCS) rats, and C3H/HeJ retinal-degeneration (*rd/rd)* mice, both widely utilized animal models in RP research, were housed and provided with standard chow and water provided *ad libitum*. Male Sprague-Dawley rats and C57BL/6 (wild type) mice were reared separately as controls. Autosomal recessive RP occurs in the RCS retina due to malfunction of MERTK, a member of the Axl subfamily of receptor tyrosine kinase, which inhibits contact between photoreceptors and the RPE [15]. Since the RPE delivers oxygen and nutrients to photoreceptors, and participates in the phagocytosis of waste material, defects in the RPE can induce retinal degeneration such as RP [16]. Photoreceptor degeneration in *rd* mice follows a pattern similar to that in human RP: apoptosis of rod photoreceptors, followed by death of cone photoreceptors [17], [18]. Rod degeneration in *rd* mice, similarly to some human RP cases, results from a mutation in the β subunit of cGMP-dependent phosphodiesterase [19], [20]. Retinal samples from RCS rats aged 5 weeks and 8 weeks, and from *rd* mice aged 3 weeks and 3 months, were prepared (n>3, respectively). Samples were obtained from both eyes so as to compare, under DIC, retinal flat-mount and histological images of retinal sections stained with Hematoxylin and Eosin (H&E). Specifically, the retina of the right eye was extracted and flat-mounted with buffer solution on slides; the left eyeball was enucleated and fixed with 4% paraformaldehyde overnight and embedded in an optimum-cutting-temperature compound. Ten-micrometer-thick sections were obtained for H&E staining.

### Automated Cell-counting Program

Based on the DIC-obtained images, we designed an automated counting program using MATLAB. Since DIC images show clearly distinctive cell boundaries and defects, no complex imaging analysis algorithm was needed. For detecting photoreceptor cells, we analyzed images obtained from DIC. Intensity within the area of cell boundary shows distinctive difference compared to other areas, and the cells show similar circle shapes. We used morphological filtering, common method for detecting circle in image processing field [21]. Morphological reconstruction was performed with repeated morphological dilation and erosion for distinctive cell boundary [22]. Those processes, also, made smoothed background of photoreceptor cells and increased contrast. Cell boundary was well recognized and detected using Canny edge detector [23]. Discontinuous lines of the cell boundary were connected with morphological dilation. Then, cell area was recognized with morphological filling. Based on the obtained image, an automated counting program was developed to automatically count the number of cells. The DIC images were taken more than 10 different positions for each state of samples and averaged number of cells was compared by Mann-Whitney U test using SPSS version 20.0 (SPSS Inc., Chicago, IL). Statistical significance was defined as P value <0.05.

## Results and Discussion

**Figure 2:**
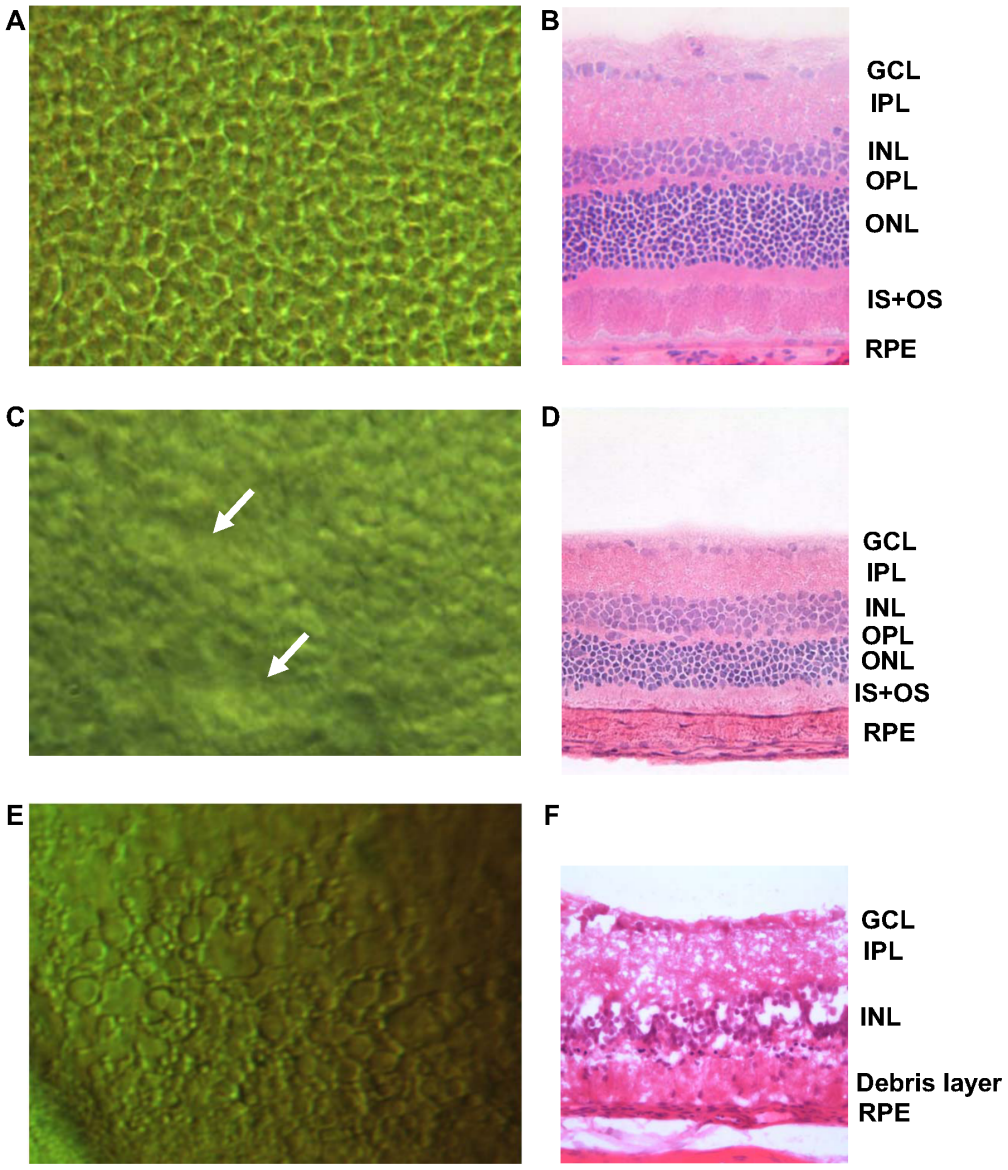
DIC microscopy and retinal-section images with progress of RP (rats). The top-left image (A) shows the normal state of the photoreceptor layer, and the top-right image (B) shows a corresponding retinal section of the specially functionalized retinal layer including the GCL, IPL, INL, OPL, ONL, IS+OS, and RPE. (C) and (D) show the states aged 5 weeks, and (E) and (F) show the states aged 8 weeks. Morphological changes with RP progression were observed in both imaging modalities.

As shown in Fig. 2A, a DIC image of a normal rat retina reveals circular cells uniformly distributed over the surface in a mosaic pattern. Each convex-shaped grain represents a photoreceptor, which are apparently separated one from another. Figure 2B shows a histological section of a normal retina without RP. The outer retina comprising the OPL, ONL, and photoreceptor layer (IS+OS) is clearly distinguishable. In order to observe the progress of the RP photoreceptors, retinal samples extracted from RCS rats aged 5 weeks were imaged (Figs. 2C, D). The DIC images show that the mosaic pattern of the photoreceptor layer is irregular and partly collapsed. The histological sections, correspondingly, show thinning and irregularity of the IS+OS, as well as a thinned ONL relative to the normal retina. The earliest histological sign of RP is known to be shortening of rod outer segments [2]. Subsequently, as RP progresses, the segments further shorten, and eventually whole cells are lost. This is reflected in reduced nuclei number and ONL thinning. The histological images of the early stage of RP, in this study, show the similar signs. Our DIC imaging could easily detect early-RP-stage photoreceptor degeneration manifesting as distinctive morphological changes to individual photoreceptors. It also reveals non-uniform photoreceptor degeneration, which is to say that certain areas degenerated first. In human RP, rod-cell loss usually initiates in the mid periphery and sectoral RP, and it has been reported that photoreceptor degeneration is limited to one or two quadrants [24]. This might explain our findings. Figure 2E, 8 weeks RP progression, shows, as a result of the severe degeneration, an irregular and amorphous pattern without the characteristic photoreceptor mosaic. Figure 2F, the corresponding histological image, shows that the entire outer retina has degenerated into a debris layer, as the previous study showed [4]. The inner-retinal GCL, IPL and INL also were disrupted.

Since DIC images are obtained by the interference of two bright-field light sources, and because phase differences are converted to visible changes, a small phase difference is sufficient for photoreceptor detection. If the phase difference attains a half or full wavelength, it can cause complete destructive or constructive interference, respectively [25], [26]. In the case of biological tissue, the phase difference is small enough, since the refractive index is similar. The thickness difference is the key factor generating interference in DIC imaging. Accordingly, disease-driven tissue-thickness changes show differences between normal and abnormal tissue states [27]. The Figure 2A DIC images of the normal state show an obvious contrast for each photoreceptor. The offset of the two beams is enough small to distinguish each cell when the cells are perfectly normal and the boundary is distinguishable. Also, the cells are in the convex shape in the normal state while cells manifesting ophthalmological disease lose tautness and collapse. The optical path difference of normal cells allows for adequate interference, in contrast to RP cells. In the present experimentation, the depths measured in moving the focal point from the upper-most to lower-most surfaces were markedly different. In the case of the normal state, the depth was around 50 µm, whereas for the RP sample it was about 35 µm. The images of the retinal sections stained with H&E show that the total thicknesses of the RP-affected tissues decreased.

**Figure 3:**
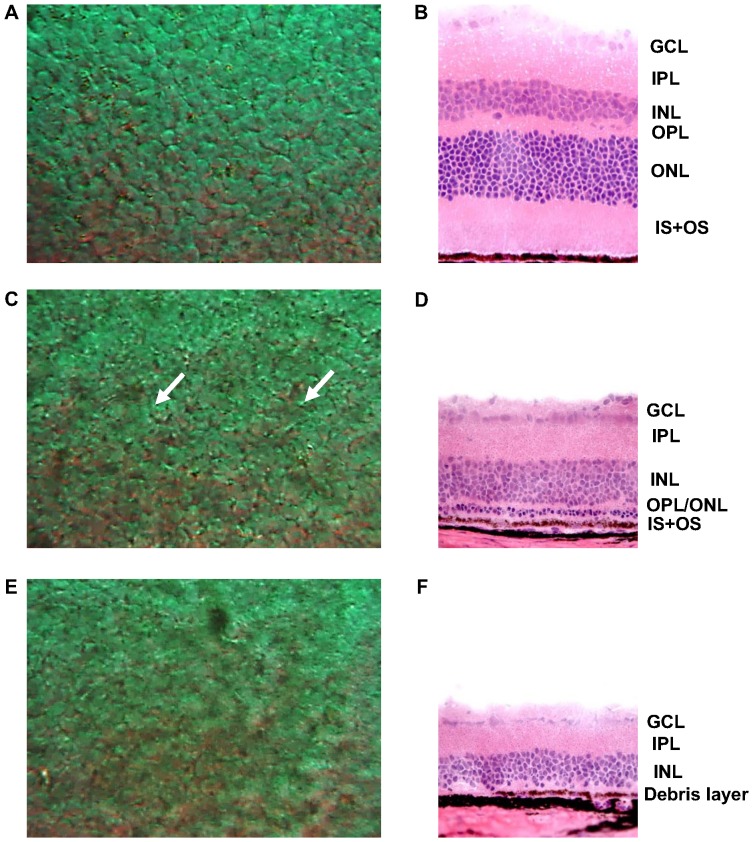
DIC microscopy and retinal-section images with progress of RP (mice). The top-left image (A) shows the normal state of the photoreceptor layer, and the top-right image (B) shows a corresponding retinal section. (C) and (D) show the states aged 3 weeks, and E and F show the states aged 3 months. Morphological changes with RP progression were observed in both imaging modalities.

The other RP model, *rd* mouse, was prepared in order to validate the results obtained with the RCS rats. Normal control mice and two different stages of *rd* mice were observed under DIC microscopy and histological sectioning. Figure 3 shows DIC and histological-sectional images of the control, *rd* mice aged 3 weeks, and *rd* mice aged 3 months. As in Figures 3A and 3B, the normal mouse retina shows photoreceptors’ mosaic pattern with distinct cell boundaries (by DIC) and intact outer retinal structure (by histological section). In retinal samples of *rd* mice aged 3 weeks (Figs. 3C and 3D), the DIC images show an irregular mosaic pattern for the photoreceptor layer, as is indicated in Figure 2C. The histological sections also show marked thinning of the ONL and IS+OS. As RP progressed to the advanced stage (Figs. 3E and 3F), the photoreceptor mosaic pattern disappeared altogether, the DIC images showing that an amorphous pattern had taken its place. According to the histological sections, the entire outer retinal layer had degenerated into a debris layer. All of these results are consistent with the RCS rat findings.

**Figure 4:**
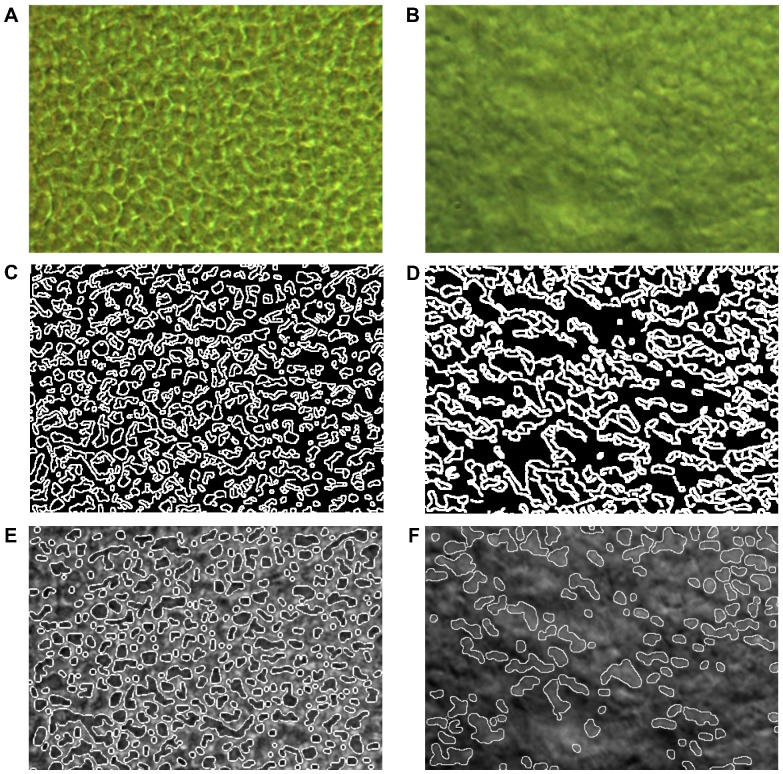
Counting analysis (rats). (A) and (B) show original DIC images of the normal and progressed RP (5 weeks) states, respectively. (C) and (D) show counted cell boundaries analyzed by MATLAB using a morphological dilation and erosion algorithm. (E) and (F) show the combined images of original and analyzed images, which indicate well-matched positions.

The DIC images were also analyzed by MATLAB for automatic diagnosis of RP based only on images [28]. This automatic counting analysis shows a possibility of drawing cell margin approximately in spited of some missing cells. Figures 4C and D show the connected edge lines, and Figures 4E and F show the detected images projected to the original images of the photoreceptor layer obtained from the RCS rats. As can be seen in Figure 4E, the cells are positioned densely and distinguishably; however, in Figure 4F, it is apparent that in the RP state, there are only a small number of cells represented, due to the degeneration of the photoreceptors. Based on the obtained images, the automated cell-counting program was designed. A total of 342 cells in the normal state were counted (Fig. 4E), whereas only 118 cells were detected in the RP state (Fig. 4F). The program counted an average of 282 cells in the other normal photoreceptor images, but only 143 cells in the images representing the RP state of the rat model aged 5 weeks (p<0.05). Images of the *rd* mouse model were analyzed according to the same cell-counting program. The number of cells of *rd* mouse shows an apparently sharp cell decrement, specifically from 255 to 170 cells (p<0.05). This RP-induced decrease agreed well with the closed-loop decremental tendency. Also, the tissue shrinkage could be confirmed by the cell decrement. A number of cells in the photoreceptor sample with RP at 8 weeks were even more decreased, since the RP had completely progressed. Thereby, the automated counting method which needs more validation in future research was demonstrated to be a possible diagnostic method for photoreceptor diseases that tend to manifest cell-number decrements.

## Conclusions

The images obtained demonstrate that by the DIC technique, we can clearly distinguish RP-affected abnormal retinal tissue from normal retinal tissue. Also, DIC images can be used to estimate the elapsed time of RP. The RCS rat and *rd* mouse images obtained using the DIC technique well coincided with the measured histological morphologies of the photoreceptors. Also, the clear DIC images made possible the application of an automated counting program (designed using MATLAB) to image analysis and photoreceptor counting. Based on our results, we conclude that the DIC technique is a feasible and effective method of RP diagnosis. Indeed, more generally, the novel application of DIC microscopy to retinal imaging at the cell level potentially can be utilized as a simple and convenient retinal-disease-diagnostic technique. Future research should focus on the potential application of DIC microscopy to *in vivo* and real-time diagnostics and monitoring of human RP.
